# At the Interface of Three Nucleic Acids: The Role of RNA-Binding Proteins and Poly(ADP-ribose) in DNA Repair

**Published:** 2017

**Authors:** E. E. Alemasova, O. I. Lavrik

**Affiliations:** Institute of Chemical Biology and Fundamental Medicine, SB RAS, Novosibirsk, 630090, Russia; Novosibirsk State University, Novosibirsk, 630090, Russia

**Keywords:** DNA repair, intrinsically disordered proteins, poly(ADP-ribose), RNA-binding proteins, Y-box-binding protein 1

## Abstract

RNA-binding proteins (RBPs) regulate RNA metabolism, from synthesis to decay. When bound to RNA, RBPs act as guardians of the genome integrity at different levels, from DNA damage prevention to the post-transcriptional regulation of gene expression. Recently, RBPs have been shown to participate in DNA repair. This fact is of special interest as DNA repair pathways do not generally involve RNA. DNA damage in higher organisms triggers the formation of the RNA-like polymer – poly(ADP-ribose) (PAR). Nucleic acid-like properties allow PAR to recruit DNA- and RNA-binding proteins to the site of DNA damage. It is suggested that poly(ADP-ribose) and RBPs not only modulate the activities of DNA repair factors, but that they also play an important role in the formation of transient repairosome complexes in the nucleus. Cytoplasmic biomolecules are subjected to similar sorting during the formation of RNA assemblages by functionally related mRNAs and promiscuous RBPs. The Y-box-binding protein 1 (YB-1) is the major component of cytoplasmic RNA granules. Although YB-1 is a classic RNA-binding protein, it is now regarded as a non-canonical factor of DNA repair.

## INTRODUCTION

DNA, RNA and poly(ADP-ribose) (PAR) are the three essential cellular nucleic acids whose functions are tightly interlinked and effected by specific mediator proteins. Some of DNA-, RNA-, and PAR-binding proteins can also interact with other types of nucleic acids distinct from their classic targets. These proteins contain a broad range of disordered regions in their structure that can accommodate any ligand upon binding. In this review, we attempt to summarize recent research findings pertaining to the interactions between the three essential nucleic acids driven by multifunctional cellular proteins. As an example, Y-box-binding protein 1 (YB-1) is discussed.

## INTERFERENCE OF DNA REPAIR AND TRANSCRIPTION

Base excision repair (BER) provides a clear picture of DNA repair and RNA metabolism coupling, since numerous molecules of this pathway, including APE1, SMUG1 and PARP1, are involved in RNA metabolism [1]. Obviously, transcription factors can mediate DNA repair by regulating the expression of repair enzymes [2]. However, the reverse is also possible: a few DNA repair enzymes may serve as transcriptional coactivators [3]. For example, thymine DNA glycosylase (TDG), which is involved in BER, is capable of activating gene transcription by recruiting coactivators [4]. The enzyme performs dynamic demethylation at promoters of silent and developmentally poised genes, as well as active gene enhancers for a rapid transcriptional response [5, 6].

DNA repair and transcription do not tend to occur simultaneously. At least, this is true for constitutively expressed housekeeping genes. Some bulky DNA damage stall RNA-polymerase II progression and trigger nucleotide excision repair (NER) (this subpathway of NER is called transcription-coupled NER (TC-NER) [7]. The mutagenic potential of other DNA lesions is minimized by inhibiting transcription at the site of a lesion; for instance, gene expression is down regulated during BER-assisted repair of oxidatively damaged DNA [8].

Signal-dependent and developmentally poised genes, on the contrary, require scheduled DNA damage to the promoter in order to trigger transcriptional activation [3]. An important regulatory mechanism for the expression of such genes is the promoter-proximal pausing of RNA polymerase II [9]. Transcription is activated, while elongation is suppressed at early time-points [10]. The escape of paused RNA polymerase II into productive elongation is mediated by DNA repair enzymes and chromatin remodeling factors. For example, the estrogen receptor activates lysine-specific histone demethylase 1 (LSD1), which demethylates histone H3. The oxidation process is accompanied by the release of a hydrogen peroxide byproduct, which converts adjacent guanines to 8-oxoguanine (8-oxoG) [11]. The repair of 8-oxoG by DNA glycosylases induces single-strand breaks that serve as entry points to DNA endonucleases, including topoisomerase IIβ [12]. When long genes are expressed, TopoIIβ creates DNA breaks not only in the promoters, but also in the reading frames, thus maintaining transcription elongation [13]. Recent findings have demonstrated that inhibition of topoisomerases suppresses the expression of long genes in yeasts [14, 15]. There is a view that the ensuing double-stranded DNA breaks relax DNA and recruit DNA damage response proteins and repair enzymes, such as PARP1 and DNA protein kinases, which leads to licensing of chromatin for transcription [12]. In human cells, DNA breaks and respective DNA repair signals are involved in the release of paused Pol II into productive synthesis and elongation of the genes that are activated following exposure to external stressors [16]. Poly(ADP-ribose) (PAR) polymerase 1 (PARP1) has been identified among the chromatin remodeling factors that control Pol II pausing. PARP1 is believed to play a role in transcription elongation due to PAR-coupled nucleosome disassembly [17]. However, poly(ADP-ribosyl)ation induced by DNA damage in the proximity of gene promoters also seems to attract the RNA-binding proteins important for Pol II docking.

Interestingly, RNA transcripts arising from a DNA lesion may trigger repair activation. It has been shown that spontaneous double-stranded DNA breaks induce ectopic transcription to give rise to short non-coding RNAs (DSB-induced small RNAs, diRNAs) 21 nucleotides long [18]. Francia *et al*. showed that diRNAs recruit enzymes to repair double-stranded breaks at the site of origin [18]. Talhaoui *et al. *have recently discovered a role for PARP1 and PARP2 in poly(ADP-ribosyl)ation of DNA strand break termini [19]. It is possible that this mechanism can contribute both to chromatin remodeling and DNA repair [19].

Some transcription factors have been shown to directly participate in DNA repair [20]. These transcription factors are thought to trigger local chromatin remodeling, thus activating DNA repair in the target sequences [21].

Collectively, transcription factors provide an extra layer of protection to the genome. Every tissue undergoes DNA damage from different sources: very high rates of oxygen metabolism in neurons lead to elevated levels of oxidative DNA lesions, whereas skin cells cope with increased UV-induced DNA damage [20]. Since transcription factors are regulated by extracellular signals and stress-activated pathways, they can confer protection to cells of a certain type [20]. Due to heterogeneous DNA repair along the genome (there is a gradient of DNA repair, with the rate decreasing towards the 3′-end of the gene), transcription factors ensure the genomic stability of the key promoter and enhancer regions of the genes being transcriptionally regulated [22].

## EUKARYOTIC "RNA OPERONS"

F. Jacob and J. Monod were the first to propose the term “operon” in 1961. According to the theory, a cluster of genes is located sequentially within an operon. The genes in the operon are together transcribed into one polycistronic mRNA, which is further translated to yield the final components of a functional complex in close proximity to each other to ensure rapid assembly. Later studies into the ribosomal profile of Escherichia coli gene expression supported this theory and demonstrated that proteins are synthesized precisely to meet the stoichiometry of the multiprotein complex [23].

**Fig. 1:**
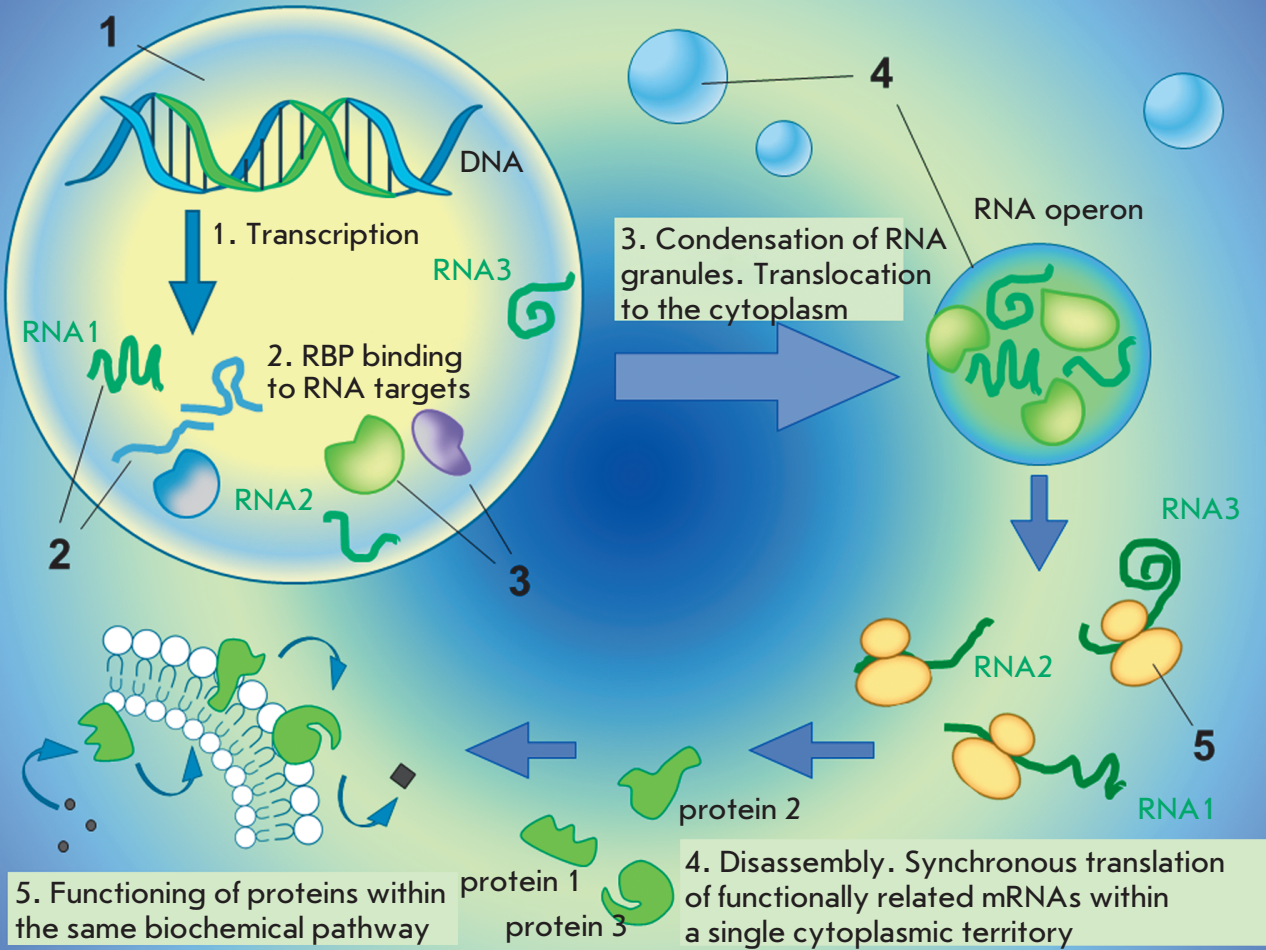
Eukaryotic RNA operons (schematic representation) 1 – nucleus; 2 – pre-mRNA; 3 – RNA-binding proteins; 4 – cytoplasmic RNA granules (RNA operons); 5 – ribosome. The figure schematically shows the formation and functioning of cytoplasmic RNA assemblages. These complexes of functionally related mRNAs and RBPs act as RNA operons that facilitate the synchronous translation of proteins involved in the same biochemical pathway

DNA operons are rare in the genome of eukaryotes, and mRNAs are mainly monocistronic. The loss of DNA operons in higher organisms could be attributed to the polar effect of nonsense mutations and the complicated regulatory network of synthesis of multifunctional proteins, which are abundant in the eukaryotic cell [24]. For this reason, the eukaryotic expression is partly regulated at the post-transcriptional level, with mRNAs that encode functionally related proteins assembling into RNA operons (*Fig. 1*), thus acquiring a common fate [25]. The principal structural and functional unit of this process is the numerous RNA-binding proteins (RBPs) that bind to RNA motifs to form ribonucleoprotein (RNP) complexes [26]. RNP complexes structurally represent the RNA operon, which allows functionally related proteins arising from different mRNAs to be jointly translated at a single cytoplasmic location [27]. The potential of RNP complexes to act dynamically and independently of the cellular environment is attributed to the mechanism called liquid demixing [28-37] that is triggered by intrinsically disordered RNA-binding proteins.

## THE NEW FUNCTIONS OF RNA-BINDING PROTEINS IN RESPONSE TO DNA DAMAGE

The recent progress achieved in research has highlighted the role that RNA-binding proteins play as guardians of genome stability [38].

DNA damage induces down-regulation of gene expression at different levels. The first step involves the suppression of transcription and pre-mRNA 3’-end processing [39, 40]. The biosynthesis of functional proteins decreases following a switch in alternative splicing from in-frame variants to variants prone to nonsense-mediated decay [41, 42]. Finally, DNA lesions affect the stability of many mRNAs [43] and inhibit translation [44, 45].

However, although the overall expression levels drop, the DNA repair machinery possesses specific mechanisms that allow it to enable the synthesis of the proteins engaged in the repair process. Suppressed translation may not affect the mRNAs that encode repair enzymes [46]. According to the model of RNA operon, mRNAs coding for functionally related proteins are together regulated at the post-transcriptional level. Overall, a single RBP such as HuR can control the expression of a broad range of genes that are involved in DNA repair [47-49].

RNA-binding proteins mediate transcription and chromatin remodeling, and they can directly participate in DNA repair [50, 51]. RBPs migrate to the sites of DNA damage [52-54], which can be explained by their ability to bind to the short non-coding mRNAs (ncRNA) that are formed at the site of a break [18, 50, 55], or by an RNA-independent mechanism.

Gene transcription at a high biosynthesis rate or with long transcripts sometimes continues into the S-phase [56], with a possibility for RNA-DNA-hybrids (R-loops), which impact the transcription and threaten genome integrity [57]. R-loop formation is prevented mainly due to RNA-binding protein-coupled packing of pre-mRNA during synthesis [58, 59].

Post-translational modification (PTM) of proteins is crucial to a cellular response to DNA damage. RBP is a primary set of proteins that are phosphorylated [60, 61] and poly(ADP-ribosyl)ated [62] under the control of DNA damage. Genotoxic stressors also trigger an increase in the levels of acetylation of certain RNA-binding proteins [63].

Finally, DNA damage facilitates the bidirectional relocation of RNA-binding proteins between the nucleus and the cytoplasm [64, 65], thus contributing to the coordinated regulation of RNA metabolism and DNA repair by multifunctional RBPs.

## RNA-BINDING PROTEINS: MODULE ORGANIZATION

The bulk of cellular mRNA is associated with RNA-binding proteins in the form of RNP complexes. Disruption of RNA granule formation results in various disorders [66, 67]. Interaction with RBPs is required for the regulation of RNA metabolism at different levels, from biosynthesis to decay. RNA-binding proteins fulfill key functions in such processes as pre-mRNA splicing [68], polyadenylation [69], transport to the cytoplasm, and translation. RBPs also have a role in the processing of non-coding RNA: the so called microRNA (miR), circular RNA (circRNA), and long non-coding RNA (lncRNA) [70-72]. Over all, RNA-binding proteins constitute an important class of post-transcriptional gene regulators.

There are a total of 1,500 RBPs known to date [73, 74]. Many RNA-binding proteins have a modular structure, in which a few basic RNA-binding domains (RBD) are arranged to accommodate a broad range of RNA sequences [75]. Certain RBDs tend to bind short sequences and display poor affinity for RNA; however, the interaction interface formed by multiple modules ensures a high affinity and specificity towards an RNA target. The superposition of weak interactions facilitates the regulation of assembly and disassembly of RNP complexes that may be mediated by an RNA-like polymer of poly(ADP-ribose) [76, 77]. Owing to the modular structure of RNA-binding proteins, different RNAs may be targeted by the same RBP [75]. A beautiful example of specific target binding promoted by tandem RBDs is the proteins of the Pumilio family (Puf), in which three amino acid side chains of each of the protein’s eight domains establish contacts with a different RNA base [78]. This “RNA recognition code” could be utilized to produce proteins with the desired binding specificity [79]. RBDs, for example, RNA-binding motif (RRM), in certain cases may also serve for protein-protein interaction [80].

It has been recently shown that besides regular RBDs, an essential role in RNA recognition is played by intrinsically disordered protein regions (IDPRs), which are highly enriched in RNA-binding proteins as compared to the total human proteome [81]. A total of 20% of mammalian proteins identified as RBPs are intrinsically disordered by over 80% [82]. Like regular RBDs, the regions with disordered sequences in RNA-binding proteins are arranged into modules that are repeated nonrandomly within a single amino acid sequence and, in some cases, may combine with globular domains [82]. Importantly, the emergence of disordered proteins in RBPs correlates with the complexity of the transcriptome in eukaryotes during evolution [83].

## DANCING PROTEINS, CHAMELEON PROTEINS, 4D AND PROTEIN CLOUDS

The new terms [84-87] coined to describe proteins without a stable 3D structure reflect the global flexibility and dynamic landscapes of intrinsically disordered proteins (IDPs) or protein regions (intrinsically disordered protein regions, IDPRs) [88]. Since the 3D protein structure is maintained by non-covalent atomic forces such as hydrogen bonding, hydrophobic interactions, van der Waals forces, etc., the intrinsic disorder, as well as the unique structure of globular proteins, is encoded by the amino acid sequence. The combination of a high net charge and low mean hydrophobicity drives the emergence of a natively unfolded protein conformation under physiological conditions [89]. The amino acid sequence of IDPs and IDPRs is enriched in Pro, Arg, Gly, Gln, Ser, Glu, Lys, and Ala but depleted in Cys, Trp, Tyr, Phe, Ile, Leu, Val, and Asn [90].

Intrinsically disordered proteins partially adopt a certain 3D structure following a change in the environment or upon binding to a ligand [91]. Their folding may also be facilitated by an elevated temperature boosting hydrophobic interactions [92], pH changes decreasing the net charge [92], as well as the presence of ions neutralizing electrostatic repulsion between clusters of amino acid residues of the same charge [93, 94]. Inside the cell, intrinsically disordered proteins adopt a rigid secondary structure after binding to ligands: small molecules, cofactors, proteins, nucleic acids, membranes, etc. [91, 95].

The functions of most proteins, in particular IDPs, are modulated through post-transcriptional modifications (PTM). As many as 300 PTMs have been identified to occur in the cell [96]. Although DNA only encodes 20 amino acids, the diversity of amino acid residues in proteins exceeds 140, owing to PTMs [97]. Proteins are mainly targeted in the disordered regions [98, 99].

IDPs and IDPR-containing proteins seem to play a central role in interactomes [100]. About 30–40% of eukaryotic proteins carry lengthy IDPRs [101], with intrinsically disordered proteins carrying out the key functions in transcription and intracellular signaling cascades [102]. In 2005, it was first suggested that hub proteins (containing multiple protein-protein interaction links within interactomes) might be enriched in IDPR [103]. Extensive studies allowed researchers to differentiate hub proteins into static and dynamic hubs [104, 105]: the former clustering into modules, which represent functional complexes with a high degree of interplay between the components (such as the transcription initiation machine), while the latter ensure interconnection of the modules [106]. IDPRs proved to be significantly enriched in dynamic hubs [107], hence elucidating the role of intrinsic disorder in guiding cellular processes [100].

IDPRs have plenty of functions. They are responsible for the autoinhibition of enzymes. In this regard, disorder-to-order transition acts as a switch on-switch off mechanism for the target protein [108]. This mechanism is employed for the activation of PARP1 during DNA repair, resulting in DNA damage signaling [109]. Another interesting example is the role of IDPR-containing proteins in protein quality control, with chaperone disorder-to-order transition being stress-induced [110]. There is data suggesting that IDPs act as molecular shields that prevent the aggregation of intrinsically disordered proteins by steric interference under stress conditions [111]. IDPRs can also regulate tissue-specific protein interactions at the transcriptional level. Buljan *et al*. [112] and Ellis *et al. *[113] showed that the enrichment of IDPRs in proteins is due to tissue-specific spliced exons [112]. Similarly, tissue-specific exons contribute to the majority of the disordered regions targeted by PTMs and motifs binding partner molecules [112]. The proteins translated from mRNAs enriched in tissue-specific exons occupy central positions in protein interaction networks and have different interaction partners in these tissues [112].

The presence of conserved IDPRs in the structure of mammalian early DNA base excision repair enzymes is a unique feature that their homologues in lower organisms do not have [114]. The IDPRs of repair enzymes are involved in DNA damage recognition, binding to interaction partners; they provide key sites for the PTMs that modulate stability, enzyme-, and DNA-binding activity, the intracellular localization of repair proteins; and they provide higher organisms with an advantage over the protein size, reducing intracellular crowding [115-119].

Finally, IDPs and IDPRs play a crucial role in the formation of dynamic macromolecular assemblages inside the cell, including RNP granules and DNA repair complexes.

## PHASE TRANSITIONS OF BIOMOLECULES

**Fig. 2:**
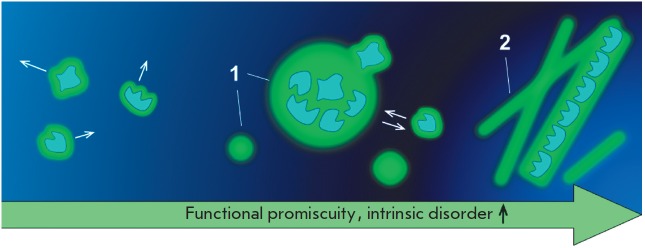
Phase transitions of biomolecules 1 – functional membraneless organelles; 2 – pathological amyloid aggregates of proteins. Cellular biomolecules undergo phase transitions as water does. In the gaseous state, biomolecules are dispersed throughout the cell and do not interact with each other. A local increase in the concentration of promiscuous and intrinsically disordered proteins results in intracellular liquid demixing and induces the assembly of membraneless compartments that have liquid-like properties [30-32]. The liquid-like state is maintained by multiple weak interactions among the interaction partners. An irreversible transition into a condensed liquid state appears to lead to amyloid fibers that are associated with such disorders as Alzheimer’s disease [35]

According to the recent findings reported in [29, 30, 33-35, 37], biochemical processes inside the cell are separated by phase transitions of biomolecules (*Fig. 2*). This paradigm states that the formation of membraneless compartments is similar to that of dispersed droplets upon emulsion breakdown (so called *liquid demixing*) [28-30, 120-122]. Intrinsically disordered proteins play a key role in phase transition events [31]. The structural plasticity and conformational flexibility of IDPs allow them to interact with multiple, structurally unrelated partners [32]. Many IDPs contain low-complexity domains (LCDs) that are prone to multimerization, driven by favorable changes in potential energy [33]. Liquid demixing results in the separation of proteins and their ligands within a compartment with a microenvironment distinct from that of other cellular plasm, thus increasing the local concentrations of interacting molecules and promoting biochemical processes [34].

The formation of RNP complexes is one of the important representations of membraneless compartmentalization by means of phase transitions of mRNA and corresponding IDPR-containing RNA-binding proteins [27]. The RNAs present in these complexes maintain their solubility [35, 36], which seems to facilitate downstream translation [27]. However, phase transitions could occur independently of RNAs only in the presence of proteins, such as in the case of formation of centrosomes (microtubule nucleation sites) [123]. Altmeyer *et al. *reported that the assembly of multiprotein repair complexes at the sites of DNA damage is achieved through liquid demixing. It was also suggested that the formation of a non-membranous DNA repair compartment also has a role in the bridging of DNA ends and their protection from nucleases [124, 125].

Phase transitions of proteins and nucleic acids to give rise to dynamic ensembles is initiated by an increase in the concentration of components, followed by self-aggregation [126], or could occur in response to changes in the microenvironment, such as pH, ionic strength, or temperature [127]. In addition, certain biomolecules are able to act as nucleation centers of multiprotein complexes, followed by separation of the intracellular plasma into two liquid phases with varying properties [37].

Single-stranded RNA [27, 128] and DNA (ssDNA) [129] are the preferred options for the nucleation of phase transition. Both biomolecules display significantly more plasticity as compared to double-stranded DNA and share such properties as a negative charge and relatively low complexity due to a limited presence of unique building units. All these features are indicative of intrinsic disorder [33]. Higher organisms reached the peak of intracellular plasma self-organization upon acquisition of a “third nucleic acid”, poly(ADP-ribose), a polymer with no ability to store information, an extremely simple structure consisting of ADP-ribose units, and a short lifetime. It is possible that poly(ADP-ribose) is the key agent in the regulation of phase transitions in the cell.

## POLY(ADP-RIBOSE) AND POLY(ADP-RIBOSYL)ATION

**Fig. 3:**
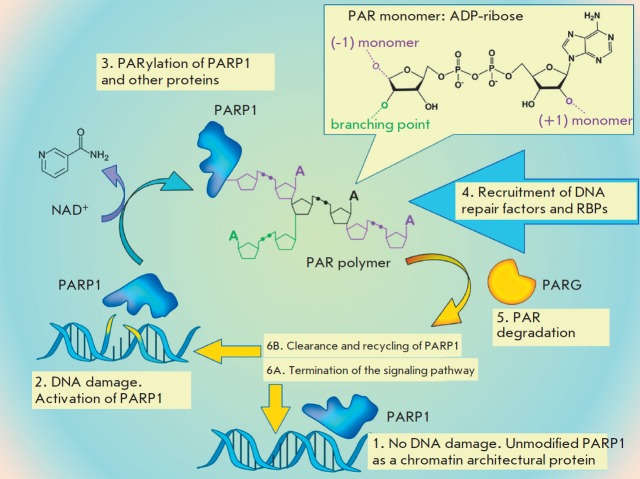
PARP1- dependent DNA damage signaling (schematic representation)

Poly(ADP-ribose) is a linear or branched polymer chain consisting of identical molecular units: monomers of ADP-ribose produced from NAD^+^ via PARP1-catalyzed PAR synthesis (*Fig. 3*) [130]. Under physiological conditions, PAR has a dynamic multiglobular structure depending on the polymer size, which allows the polymer to fit the structure of the bound ligand [131]. Adenine residues in PAR, identically to those in nucleic acids, adopt an anti-conformation that is capable of base stacking and formation of hydrogen bonds [132]. The secondary structure of PAR as a helix, which has been confirmed *in vitro *by spectral analysis [133], can occur at high ionic strength (4 M NaCl) or upon binding to proteins under physiological conditions [132]. The PAR polymer carries two negatively charged phosphates in each monomer (ADP-ribose unit), while RNA and ssDNA only carry one negative charge per unit [134]. In the absence of genotoxic stressors, intracellular PAR levels are very low and ADP-ribose exists in a relatively stable state of monomers and oligomers (half-life t_1/2_ ~7.7 h). Extensive local biosynthesis of a very short-lived PAR polymer (t_1/2_ less than 1 min) is triggered by DNA damage [135-137]. The prominent feature of poly(ADP-ribose) is its involvement in post-translational protein modifications.

By analogy with DNA and RNA, the enzymes that catalyze the synthesis of PAR are called PAR polymerases (PARPs). The human PARP family includes 17 members with similar catalytic domains [138]. Only four members are capable of catalyzing PAR synthesis: PARP1, PARP2, and two tankirases [138, 139]. PARP1 and PARP2 act as guardians of genome integrity [140]. Tankirases synthesize linear PAR chains up to 20 monomers long [141]. Their functions are exerted when the spindle apparatus begins to form [142]. Tankirases also control centrosome functions [143].

PARP1 is activated upon binding to exposed bases on the loose ends of DNA breaks [144]. Recognition of a DNA lesion induces conformational changes in the autoinhibitory domain of PARP1, which locally unfolds, thus ceasing to interfere with NAD^+^ binding in the active center [109]. As a result of intermolecular rearrangement of PARP1 attracting the catalytic domain to the damage site, the automodification domain is positioned close to the active center open to modification by PAR [145]. This finding provides insight into why PARP is the preferred target for poly(ADP-ribosyl)ation [134]. The PAR acceptor amino acid residues identified in PARP1 and other poly(ADP-ribosyl)ation targets to date are multivarious: Lys, Arg, Glu, Asp, Cys, Ser, Thr, Sep (through the phosphate group) and Asn, although charged amino acid residues are typically responsible for this function [146-149]. Bearing in mind that the rate of PAR biosynthesis is limited to NAD^+^ breakdown, it is tempting to suggest that the binding of ADP-ribose to a target protein in the presence of activated PARP1 occurs via any amino acid residue exposed on the protein surface [125]. Specific PAR-mediated modulation of cellular processes can be achieved through different local microenvironments of PARP1 and its ligand, rather than through specific PAR acceptor sites in the target protein [125].

PAR binds non-covalently to many proteins. Among the proteins associated with PAR and/or prone to this PTM are certain repair enzymes, chromatin remodeling proteins, RNA-binding proteins, and transcription factors [62, 150]. Numerous functions exerted by PAR in the cell are implemented via dynamic interactions between poly(ADP-ribose) and PAR-binding proteins. Protein relocation caused by local synthesis of PAR influences cellular signaling, DNA damage response, transcription regulation, protein stability, and cell fate [151]. Several PAR-binding modules have been described; their structure varies from completely ordered domains to intrinsically disordered regions capable of forming multivalent contacts with the PAR polymer [125].

PAR can also be recognized by RNA- and DNA- binding motifs [125]. Since not only specific interactions but also dynamic changes in the concentrations of interacting molecules influence macromolecular ensembles, PAR may outcompete RNA binding of RBPs at the peaks of PARylation, resulting in RBPs relocalization to DNA damage sites [152]. The DNA-binding domains of DNA repair enzymes and transcription factors may also facilitate the recruitment of these proteins to the DNA damage sites in a PAR-dependent mechanism [153, 154].

It has been recently shown that PAR can nucleate the intracellular phase transitions of such RNA-binding proteins as FUS (TLS), EWS (EWSR1), and TAF15 at microlaser-generated sites of DNA lesions [124]. Intracellular compartmentalization initiated by PAR-dependent phase separation can underlie the mechanisms by which poly(ADP-ribose) is involved in DNA- and RNA-dependent cellular events: for example, the formation of stress-granules [155], nucleoli [156], spliceosomes [157], and transcriptosomes [158]. In the event of transcription regulation, the phase transition of FUS (TLS), EWS (EWSR1), and TAF15 at gene promoters appears to create sites for the binding of the C-terminal disordered domain of RNA-polymerase II [159]. PARylation in close proximity to promoters seems to facilitate transcription, especially if keeping in mind that DNA breaks in promoters and reading frames may be scheduled [5, 13, 17].

Long-lived PAR carries such risks as stripping RNA-and DNA binding proteins off their ligands, phase transitions of dynamic droplets into the insoluble protein aggregates found in pathological states [33], as well as the energy crisis arising from depleted NAD^+^ pools [160]. That is why PARylation is subjected to tight control by the enzymes that break down PAR and remove ADP-ribose residues from modified proteins [161]. The key ADP-ribose-degrading enzyme is poly(ADP-ribose)glycohydrolase (PARG), which exhibits endo- and exo-hydrolase activities; the latter activity being dominant over the first one [162]. Since degradation occurs when the polymer is available, PAR-binding proteins can potentially counteract PARG. PARG is actually unable to cleave the proximal ADP-ribose monomer, which appears to be due to steric hindrance [163]. ADP-ribose units are removed from mono(ADP-ribosyl)ated proteins by specific enzymes [164]. Dynamic regulation of PAR levels may provide a physiological balance between DNA- and RNA-protein interactions in different cellular contexts.

## Y-BOX-BINDING PROTEIN 1

The Y-box-binding protein 1 (YB-1) is an example of a multifunctional protein acting at the “interface of three nucleic acids.” While binding to DNA [165, 166], YB-1 carries out its functions in transcription [167] and likely in DNA repair [166, 168]. YB-1, as a transcription factor, controls the expression of stress-induced genes and the genes involved in DNA repair [167, 169, 170]. As an RNA-binding protein [167, 171], YB-1 mediates pre-mRNA splicing, is one of the major proteins constituting RNP granules in the cytoplasm [172], and modulates mRNA translation [167, 173]. There is evidence that YB-1 interacts with multiple noncoding RNAs [174, 175] and exhibits strong affinity for damaged DNA and RNA [166, 168, 176], as well as PAR-binding properties [150]. Genotoxic stress induces a relocation of YB-1 from the cytoplasm to the nucleus [177-180]. Under certain conditions, this stress-induced trafficking occurs following a specific post-translational modification of YB-1 – partial proteolytic cleavage by the 20S proteasome [181].

The bulk of the YB-1 structure is natively unfolded [167], which facilitates interaction promiscuity and confers the ability to self-aggregate, allowing for multimerization in the presence of RNA and DNA [182] or the formation of amyloid fibrils at a high ionic strength [183]. YB-1 binds to a wide range of DNA repair enzymes: base excision repair enzymes (NEIL2 [177], APE1 [184], DNA polymerase β [177], DNA polymerase δ [185], PCNA [186], DNA-ligase IIIα [177], NEIL1, PARP1, and PARP2 [187]), mismatch repair enzymes (MSH2 [185]), and DNA double-stranded breaks repair enzymes (Ku80 [185]). YB-1 is required for the recognition of bulky lesions by NER factor XPC-HR23b [188] and modulates the activity of key and regulatory BER enzymes [177, 187, 189-191].

**Fig. 4:**
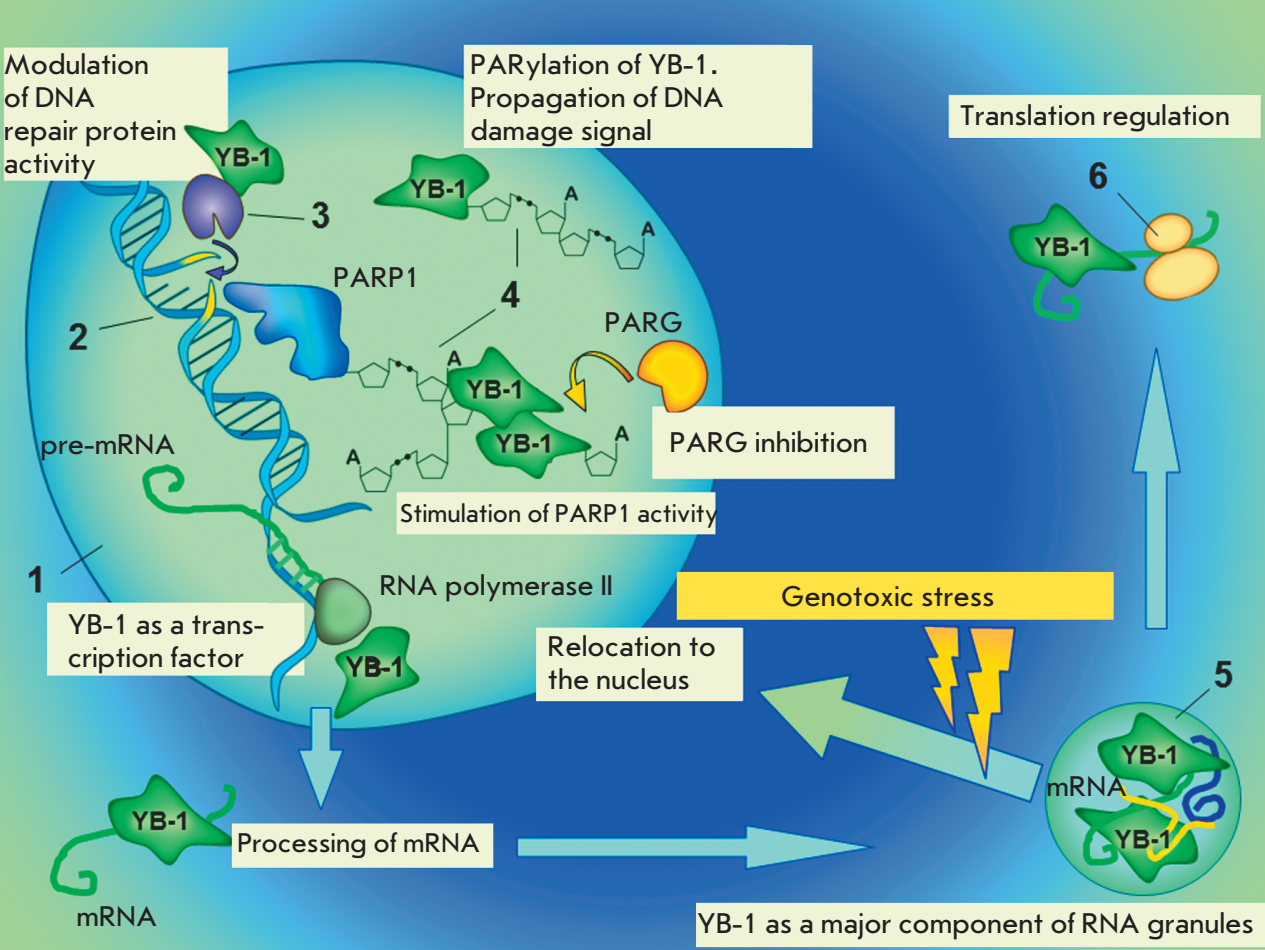
Switching of YB-1 cellular functions upon genotoxic stress (schematic representation) 1 – nucleus; 2 – DNA damage; 3 – DNA repair enzyme; 4 – poly(ADP-ribose); 5 – cytoplasmic RNA granule; 6 – ribosome

YB-1 is found in stress granules [192], is necessary in centrosome formation [193], and has a potential role in nucleolar disassembly [194]. The emergence of these membraneless compartments, as well as the formation of repair complexes at sites of DNA lesions, is orchestrated by poly(ADP-ribose) [155, 156, 195]. Recent findings have demonstrated that YB-1 is able to modulate PAR biosynthesis depending on the level of DNA damage [187] and acts as a target for poly(ADP-ribosyl)ation [187, 196]. Another feature is the fact that YB-1 protects PAR from cleavage by PARG, extending the half-life of the polymer [187]. *Fig. 4* schematically depicts the role played by YB-1 in PAR and RNA metabolism.

Over all, a transcription factor and one of the key RNA-binding cytoplasmic proteins, YB-1 display a plethora of additional functions that come into play under genotoxic conditions. Besides transcriptional and post-transcriptional regulation of gene expression, the functions of YB-1 may include participation in DNA repair and regulation of repair complex formation through PAR-dependent phase transitions of intrinsically disordered proteins and DNA repair factors enriched in IDPRs. YB-1 represents a possible pathway in which RBP may act as an extra guardian of genome integrity under stress conditions.

## CONCLUSIONS

It appears that, the higher the level of an organism, the higher is the organizational complexity of its regulatory pathways. At the same time, the limited size of the cell prompts proteins to assume a multifunctional role. The multifunctionality, i.e., the ability to assume different functions, is closely linked to the ability to have many interaction partners whose structure in most cases is determined by the function performed by a protein in the cell. A modular structure that provides a variable degree of specificity cannot solve this problem, because the number of possible interactions remains limited. This limitation is beautifully addressed by reducing the information volume of the primary structure of nucleic acids and proteins. V. Uversky [88, 197] conclusively demonstrated that a reduced protein sequence leads to the maximum possible structural complexity. The occurrence of natively unfolded proteins dramatically expanded the range of intracellular interactions due to the unique features of this protein kingdom [197]. The intrinsic multivalence and their small size render these proteins instrumental in a variety of cellular processes and make them central players in interactomes, thus acting as key regulators of protein networking.

Along with the emergence of new functions in the proteome during evolution, higher eukaryotes have developed a wide array of noncoding nucleic acids that regulate basic RNA- and DNA-protein interactions. The maintenance of genome integrity, particularly, depends on the “third nucleic acid,” poly(ADP-ribose), generated from NAD^+^ in the presence of DNA damage. PAR formation, which modulates the interactions between RNA- and DNA-binding proteins and their targets, leads to the assemblage of functional complexes. These functional assemblages are required to regulate the key processes that take place in cellular metabolism under stress conditions.

## Abbreviations

BER: base excision repair
IDP: intrinsically disordered protein
IDPR: intrinsically disordered protein region
LCD: low complexity domain
PAR: poly(ADP-ribose)
PTM: posttranslational modification
RBD: RNA-binding domain
RBP: RNA-binding protein
RNP: ribonucleoprotein
